# The effect of age on melanoma incidence and prognosis

**DOI:** 10.18632/aging.204653

**Published:** 2023-08-17

**Authors:** John F. Thompson, Gabrielle J. Williams

**Affiliations:** 1 Melanoma Institute Australia, The University of Sydney, Sydney, NSW 2060, Australia

**Keywords:** melanoma, incidence, age, prognosis

Data from around the world consistently show that the incidence of invasive melanoma increases with advancing age (Figure 1A); it is rare in children and uncommon in adolescence [1] but its incidence rises steadily thereafter. The rate of increase with age varies from country to country, but in all of them the upward trend is readily apparent. Likewise for melanoma mortality, as age increases so too does the rate of death from melanoma (Figure 1B). The rising incidence of melanoma with increasing age is shared with other common cancers such as colon and lung cancer, but the upward trajectory of incidence begins at a much younger age (Figure 1A).

**Figure 1:**
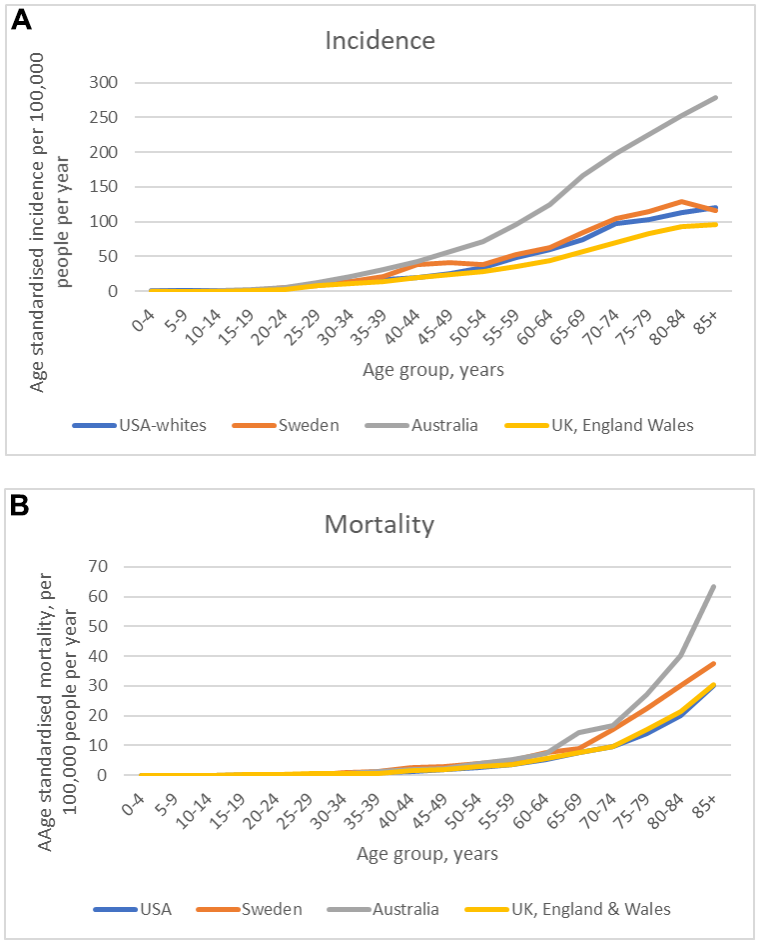
Melanoma incidence (**A**) and mortality (**B**) according to age [2].

While invasive melanoma is the focus of this discussion, the incidence of non-invasive “melanoma *in situ*” (MIS) also increases steadily with age but does not lead to the mortality associated with invasive melanoma. The most common form of MIS is lentigo maligna (LM, formerly known as Hutchinson’s melanotic freckle); its development is attributed to chronic, accumulated exposure to ultraviolet light, which is proposed as the primary reason for the increase in cases with age. The rate of progression from LM to invasive lentigo maligna melanoma (LMM) has been estimated to be 3.5% per year, with an average time for LM to progress to LMM of 28.3 years [2].

Older patients are more likely to have an uncommon subtype of invasive melanoma known as desmoplastic melanoma. Like MIS, this form of melanoma is strongly associated with chronic sun exposure and most frequently occurs on the skin of the head or neck. Desmoplastic melanomas have a somewhat higher rate of local recurrence (6%-15%) than non-desmoplastic melanomas (<5%) [3].

The great majority of invasive melanomas in elderly patients are non-desmoplastic, however, and are more likely to show features associated with poor prognosis than desmoplastic melanomas. Specifically, the melanomas are more frequently ulcerated, have a higher mitotic rate and an increased Breslow thickness, all of which are associated with worse outcomes [4]. Increasing age is associated with a greater risk of local site recurrence, distant metastasis and death from melanoma even when other risk factors have been taken into consideration [5]. However, this is not the case for older patients who present with thin melanomas (≤1.0mm in Breslow thickness), in whom the risk of regional recurrence or metastasis to distant sites does not appear to be increased, once other risk factors have been accounted for.

A patient who presents with a primary melanoma that has high risk features such as those described above is usually offered a sentinel lymph node biopsy (SLNB). Pathological examination of the SLN indicates whether metastatic melanoma cells are present in the regional lymph nodes, another important prognostic factor and increasingly the basis for selection for adjuvant post-operative treatment with newer systemic therapies.

There is a decreasing likelihood of a positive SLNB result as age increases [6]. Conversely, the likelihood of death due to blood-stream spread of melanoma to distant sites increases with advancing age. The reasons for these phenomena are largely unknown. Proposed explanations include the changes that occur in cells with aging (senescence), reducing their ability to travel through lymphatic channels to lymph nodes. Another theory suggests that degradation of the extracellular matrix that occurs with aging changes the way that cells can migrate through the lymphatic system. Yet another possibility is that anatomical and physiological changes occur in lymph nodes as age progresses, rendering them more “porous” and allowing metastatic melanoma cells to pass through them, continue with re-circulating lymph into the systemic venous system, and thence travel to distant body sites.

Currently, SLN-positive melanoma patients can be offered adjuvant systemic immunotherapy or targeted therapy. Immunotherapy agents include ipilimumab, a monoclonal antibody against cytotoxic T-lymphocyte-associated antigen 4 (CTLA-4), and pembrolizumab or nivolumab, antibodies against programmed cell death ligand 1 (PD1). These agents bind to the CTLA4 or PD1 proteins and reduce their function. Our knowledge of how expression of CTLA4 and PD1 on cells changes with age is imperfect, and how aging patients might respond to these immunotherapy agents was not well understood when clinical trials commenced. Early trials excluded patients <18 years of age and had few participants >70, but analyses within the age groups that did receive treatment suggested that efficacy was not age-related. Subsequently, non-randomized studies of both types of agents have shown that their efficacy in elderly patients was quite similar to their efficacy in younger age groups, and that adverse event profiles were also similar [7]. At the opposite end of the age spectrum, little is currently known about the efficacy of these immunotherapy agents in children and adolescents.

An alternative or additional option for systemic therapy in SLN-positive melanoma patients is targeted therapy. Overall, approximately 50% of melanomas carry a mutation in the BRAF gene, most involving a change at codon 600. This led to the development of specific BRAF inhibitors, vemurafenib and dabrafenib. Response rates for these two agents are around 50%-80%. Unfortunately, however, patients who respond to BRAF inhibitors tend to develop resistance within 6-8 months, and combination with a MAP kinase inhibitor such as trametinib is now frequently used to enhance and/or prolong response. Melanomas in younger patients are much more likely to be BRAF-positive than those in older patients (around 70% BRAF-positivity for patients <45 years has been reported but only 30% BRAF-positivity for those >70 years) but the response to BRAF inhibitors +/- a MEK inhibitor in patients >75 appears to be very similar to that in younger cohorts. For patients of all ages adverse events occur quite frequently, but can usually be managed with dosage adjustments [8]. As with immunotherapy, the efficacy of targeted therapies in children and adolescents is poorly documented.

Radiation therapy has in the past been an important component of management for older patients with melanoma brain metastases, often because surgical excision was considered inappropriate. Whole-brain radiotherapy (WBRT) was used principally to treat multiple metastases whereas stereotactic radiotherapy (SRS) was used as an alternative to surgical excision to ablate specific tumors. Both WBRT and SRS in cancer patients 70-90 years of age have been shown to be as effective as in younger age groups.

With the introduction in the past decade of effective immunotherapy and targeted therapies there is now the option to combine localized radiation with systemic therapy. The efficacy of using this strategy for melanoma brain metastases is not yet clear, but in studies to date age was not a significant factor influencing survival. It would thus appear that combination radiation therapy and immunotherapy or targeted therapy could be offered to older patients with the expectation of achieving benefits similar to those obtained in younger patients.

## CONCLUSIONS

The likelihood of developing a melanoma, whether non-invasive or invasive, increases with age and patterns of the disease differ in older age groups, with progressively higher rates of metastasis and death due to melanoma as age increases. However, it would appear that the standard existing forms of melanoma management – surgery, radiation therapy and new systemic drug therapies – are as effective and safe in older patients as they are in younger age cohorts.

## References

[1] El Sharouni MA, et al. J Am Acad Dermatol. 2023; S0190-9622(23)00440-1. 10.1016/j.jaad.2023.03.008 36935017

[2] Heenan P, et al. Lentigo Maligna. In: LeBoit PE, Burg G, Weedon D, Sarasin A, eds. World Health Organisation Classification of Tumours Skin Tumours. Lyon, France: IARC Press. 2006.

[3] Hughes TM, et al. J Eur Acad Dermatol Venereol. 2021; 35:1290–8. 10.1111/jdv.17154 33544941

[4] Balch CM, et al. J Clin Oncol. 2001; 19:3635–48. 10.1200/JCO.2001.19.16.3635 11504745

[5] El Sharouni MA, et al. Br J Surg. 2021; 108:550–3. 10.1002/bjs.11946 34043770

[6] Balch CM, et al. Ann Surg Oncol. 2014; 21:1075–81. 10.1245/s10434-013-3464-x 24531700 PMC4121329

[7] Howell AV, et al. J Geriatr Oncol. 2022; 13:1003–10. 10.1016/j.jgo.2022.05.005 35660090 PMC12507325

[8] Kohtamäki LM, et al. Melanoma Res. 2021; 31:218–23. 10.1097/CMR.0000000000000727 33675299

